# Arthroplasty for femoral neck fracture of the elderly: a narrative review

**DOI:** 10.1530/EOR-2024-0211

**Published:** 2026-07-01

**Authors:** Gautier Beckers, Maximilian Lerchenberger, Wolfgang Böcker, Boris Michael Holzapfel, Jörg Arnholdt, Dominic Simon

**Affiliations:** Department of Orthopaedics and Trauma Surgery, Musculoskeletal University Center Munich (MUM), University Hospital, LMU Munich, Munich, Germany

**Keywords:** displaced femoral neck fracture, hip hemiarthroplasty, total hip arthroplasty, elderly

## Abstract

- This review provides an up-to-date, focused, and evidence-based treatment strategy addressing the ongoing debates in the literature surrounding arthroplasty for displaced femoral neck fractures in the elderly.
- Every effort should be made to perform surgery within 24–36 h of admission. After-hours surgery may be performed safely when an experienced team and adequate resources are available; if not, postponing to daytime hours to optimize surgical conditions is reasonable.
- While pre-fracture mobility, independence, and comorbidity are relevant in treatment planning, current high-level evidence does not support a consistent clinical benefit of total hip arthroplasty over hemiarthroplasty, even in selected, higher-functioning patients.
- Given the substantially increased risk of periprosthetic fracture associated with cementless fixation, cemented stems should be preferred in elderly patients with displaced femoral neck fractures.

## Introduction

Over the past three decades, the incidence of fall-related hip fractures has shown a noticeable upward trend (1).

Moreover, femoral neck fractures (FNFs) predominantly affect women and individuals aged over 70 (2). In the coming decades, Western populations are projected to shrink while simultaneously aging and the demand for acute hip arthroplasty procedures is expected to continue to increase (3).

While the treatment of FNF in younger patients is generally more uniform, the management of displaced FNF in the elderly remains associated with considerable variation in treatment approaches between countries (4).

Although international guidelines and systematic reviews largely agree that cemented hemiarthroplasty (HA) represents the standard treatment for most elderly patients with FNF, several aspects of surgical management remain debated, including the optimal timing of surgery such as within 24 versus 48 h, the choice of surgical approach, implant selection between HA and total hip arthroplasty (THA), the role of preexisting osteoarthritis in guiding this choice, and the use of cementation.

The aim of this narrative review was to provide an overview of the existing debates in the literature surrounding FNF in the elderly and an evidence-based treatment strategy.

### Time to surgery

Regarding the timing of surgery, the orthopedic and trauma surgery community generally agrees that it should be performed as soon as possible and under optimal conditions. However, debate remains: is it preferable to undergo surgery at night by a general orthopedic team rather than waiting until the next day for a specialized team? Even if the team is specialized, should every effort be made to perform the surgery as quickly as possible, even if it means operating at night? The answers to these questions remain debated.

While ultra-early surgery (<6 h) does not appear to confer significant benefits, delayed surgery (>48 h) seems to be detrimental (5, 6).

The HIP ATTACK randomized controlled trial compared accelerated surgery (median time from fracture to surgery <6 h) with standard of care (median time from fracture to surgery <24 h) and concluded that accelerated surgery did not significantly reduce the risk of mortality or major complications compared with standard care (5). Nevertheless, reduced risk of delirium, urinary tract infection, pain, and length of stay were observed in the ultra-early surgery group (5).

Having that in mind, what is the deadline we should not exceed? Surgical delays exceeding 36 h were linked to a 2.9% increase in mortality rates, with the impact of delayed surgery becoming more pronounced in patients with higher American Society of Anesthesiologists (ASA) grades (7). The authors concluded that, in patients with ASA ≥ 3, the lowest mortality rates were observed when surgery was performed between 12 and 24 h (7). This is consistent with data from a large Swedish nationwide cohort of approximately 60,000 patients, which suggested a more patient-specific approach to surgical timing (8). In that study, surgery beyond 24 h was associated with increased mortality primarily in patients with ASA 3 and 4, although the absolute differences in mortality risk were small (8). A meta-analysis including 521,857 hip fractures reported reduced mortality when surgery was performed within 24 h compared with 24–36 h. However, it should be noted that this analysis included all surgically treated proximal femoral fractures and was not specific to arthroplasty procedures (9).

Others reported that for every 10 h delay from admission to surgery, 1-year mortality increases by approximately 5% (10) and that 2-year mortality is higher when surgery is performed more than 72 h after admission (15% for surgery within 24 h versus 21% for surgery after 72 h) (11).

In a meta-analysis comprising over 190,000 patients, Moja *et al.* found that surgical delay was associated with a heightened risk of mortality and pressure sores (6).

While ultra-early surgery does not appear to confer significant benefits, the consensus is that delaying surgery can be detrimental.

Moja *et al.*, similar to the National Institute for Health and Care Excellence (NICE) guidelines, concluded that patients with FNF should undergo surgery within 1–2 days (6), which seems to be the adequate window (12).

However, to achieve surgery within 48 h of admission, after-hour surgery is often necessary. Nevertheless, the absence of specialized operating room staff during those times is a significant concern (13).

A study by Lim *et al.* found no difference in the rates of adverse events between patients who underwent surgery during ‘opening hours on weekdays’ and those who underwent surgery ‘after hours or on weekends’, provided that staffing was adequate (14). Other concluded that the risk of adverse events was similar in patients undergoing hip fracture surgery during normal hours or after hours (15).

These conclusions need to be interpreted with caution. Most studies analyze hip fractures, including pertrochanteric fractures, rather than specifically focusing on FNF requiring joint arthroplasty (15, 16). Furthermore, although HA does not require acetabular preparation and is therefore technically less demanding, conclusions regarding the safety of after-hours procedures frequently fail to differentiate between HA and THA. Moreover, regarding the safety of overnight and after-hours procedures, simpler cases that are more accessible to less experienced surgeons and healthier patients may be preferentially selected, potentially introducing selection bias.

### Surgical approach

Each surgical approach has its own advantages and disadvantages.

Registry studies and prospective cohort analyses consistently demonstrate that surgical approach is a major independent determinant of dislocation risk after HA and THA for FNF (17, 18). Posterior approaches are associated with significantly higher dislocation rates compared with anterolateral or direct lateral approaches.

A meta-analysis comparing various surgical approaches for HA in FNF revealed that the posterior approach is associated with a higher risk of dislocation and reoperation compared with the lateral or direct anterior approach (18). With bipolar HA, the posterior approach is linked to an eight-fold rise in the risk of dislocation compared with the lateral approach (19), and a similar observation was demonstrated for THA (19).

This likely explains why the NICE guidelines discourage the posterior approach for HA (12). These findings require cautious interpretation. For instance, variations in the posterior approach (piriformis sparing or not) should be considered as it could mitigate that risk (17, 20).

The lateral approach remains widely performed, with the literature suggesting favorable results, particularly with respect to lower dislocation rates (17, 21). While this approach offers certain advantages, it is also associated with a risk of abductor mechanism insufficiency (22). In the authors’ opinion, this potential impairment of the abductor mechanism should be carefully considered when selecting the surgical approach.

Furthermore, altered gait patterns and abductor weakness may be underestimated in large registry studies, as these outcomes are not routinely captured and do not typically lead to revision in the same way as complications such as dislocation or periprosthetic fracture. Consequently, registry data may incompletely reflect functional differences between approaches.

A meta-analysis including 1,604 patients undergoing HA suggested that the direct anterior approach was associated with improved early postoperative functional outcomes, lower dislocation rates, and fewer overall complications compared with other surgical approaches (23). However, the DAA comes with a prolonged learning curve (24).

Finally, the surgeon’s experience with the chosen approach should also be considered, as it may significantly influence the outcomes associated with each of the approaches mentioned above (25).

### THA versus HA

Of all the questions in treating FNF in the elderly, perhaps the most debated and important is the choice between THA and HA, as both have benefits and drawbacks (26). From the literature, it is evident that THA is linked to longer operating times and higher dislocation rates (27, 28, 29) compared with HA. However, THA also yields superior patient-reported outcomes (27) and lower reoperation rates compared with HA (28).

Most of the debate in that area is regarding patient selection as the surgeons must consider various factors. Age, life expectancy, and activity level are pivotal elements that are closely interrelated and significantly influence the outcome. Linked to the latter consideration is whether patients can benefit from the functional advantages of THA, despite the potential for increased complications compared with HA (28, 29, 30).

Referring to age is quite straightforward, but it is crucial to also consider the patient’s profile.

To aid in the decision-making process, the activity level can be easily assessed preoperatively using user-friendly scores, such as the Parker Mobility Score (31). A score greater than 5 on this scale can indicate an active patient. Other tools, such as the Katz index of independence, evaluate a patient’s level of autonomy by assessing their performance in activities of daily living, such as bathing, dressing, toileting, transferring, continence, and feeding. Each activity is scored on a scale of 0–1, with 1 indicating that the task was performed without any assistance and 0 indicating that assistance was required. A score of 2 or less indicates full dependency, 4 suggests moderate impairment, and 6 suggests full independence (24).

**Figure 1:**
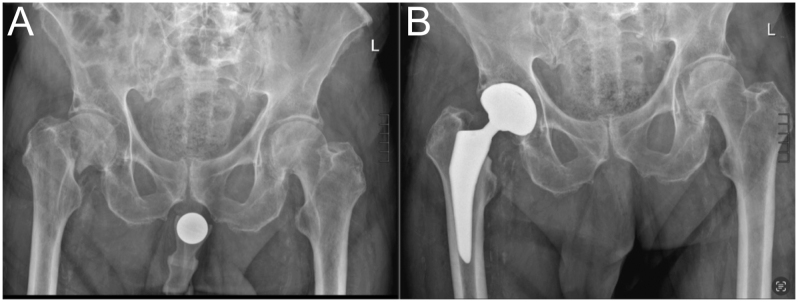
(A) Preoperative and (B) postoperative anteroposterior radiographs of the pelvis of an 86-year-old male who presented with a displaced FNF. The patient is active and independent, with a Parker score of 9. The cortical thickness index is 0.5, and the femur is classified as Dorr type B. An uncemented THA was implanted.

Studies demonstrated that both patients under 75 years and active independent patients with HA face an elevated risk of conversion surgery, attributable to factors such as acetabular erosion and pain (32, 33), likely due to higher activity levels leading to more wear, when compared to older patients (32). Furthermore, at 7–10 years of follow-up, THA in independently mobile patients demonstrates lower mortality rates and reduced morbidity compared with HA (33). Therefore, for active patients even if over 75, some authors advocate for THA (Fig. 1) (34).

**Figure 2:**
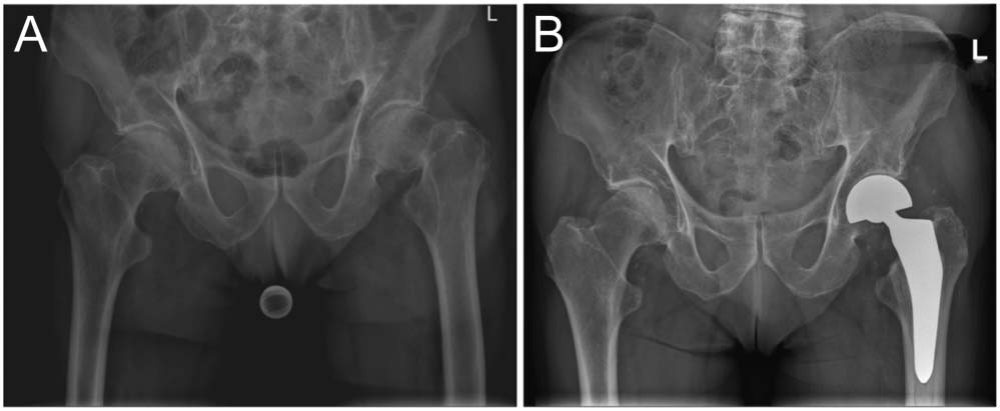
(A) Preoperative and (B) postoperative anteroposterior radiographs of the pelvis of an 83-year-old male who presented with a displaced FNF after a fall from his height. The patient is not active but lives independently with his wife at home. The cortical thickness index is 0.45, and the femur is classified as Dorr type B. There were no reports of pre-traumatic groin pain. A cemented bipolar hemiarthroplasty was implanted.

On the other end of the spectrum, patients with dementia, reduced mobility, poorer overall health, and low-demand patients (Fig. 2), could continue to benefit from HA over THA as they may undergo fewer conversions (32, 35). Furthermore, this type of patients might benefit from shorter surgeries with reduced blood loss (26, 36).

The Abbreviated Mental Test Score (AMTS) is another commonly used tool (37) to help identify patients with cognitive impairment. Patients with cognitive impairment are at a heightened risk of dislocation, particularly following THA (38). However, a study indicated that this elevated risk of dislocation can be mitigated by employing dual mobility THA, without exacerbating mortality rates compared with HA (39).

Finally, frailty, a clinical condition marked by increased vulnerability due to an age-related decline in physiological reserves, can be measured preoperatively (40). While frail patients have a higher mortality rate than non-frail patients for both HA and THA for FNF, the mortality increase was two-fold for HA and four-fold for THA. This led the authors to suggest that in frail patients, implanting an acetabular component might add complications, making HA a potentially better-tolerated option (41).

Selvam *et al.* found, in a retrospective study analyzing the preoperative factors’ influence on the decision between THA and HA for FNF, that patients with a Katz-index of 1–4 were more likely to have a HA (42). Furthermore, patients with ASA 1 and 2, indicating lower comorbidities, were more likely to receive a THA for an FNF.

The presence of pre-traumatic osteoarthritis is another subject of considerable debate. Some argue that preoperative radiographic osteoarthritis should be viewed as a contraindication to HA (33, 43) due to concerns regarding potential acetabular erosion (32), which could lead to unfavorable long-term results for osteoarthritis patients undergoing this procedure (44). However, others have found no significant difference in functional outcomes and revision rates between patients with radiographic evidence of osteoarthritis (Kellgren and Lawrence grade 3–4) and those without (Kellgren and Lawrence grade 0–2), although these findings were based on a relatively short follow-up period of 12 months (45).

It is important to note the weak association between knee pain and radiographic osteoarthritis (46), a phenomenon that may also be present in the hip, underscoring the need to distinguish between symptomatic pre-traumatic osteoarthritis and radiographic osteoarthritis in clinical decision-making.

While not accounted for following NICE guidelines (12), preexisting and symptomatic hip osteoarthritis is incorporated into the indication profile outlined in the American Academy of Orthopaedic Surgeons (AAOS) guidelines when deliberating between THA and HA for FNF (47). Further studies are warranted to explore the potential benefits of THA in FNF on symptomatic osteoarthritic hips, even in patients with limited life expectancy.

Finally, additional factors influencing the conversion rate include high BMI (32, 48), which contributes to increased wear rates due to higher joint reaction forces, and the utilization of bipolar implants (compared with unipolar) (32), which can be considered when choosing between hip HA and THA. Nevertheless, high BMI primarily increases the risk of infection and medical complications (49).

Although the debate regarding potential advantages of THA in displaced FNF continues, higher-quality randomized trials and contemporary meta-analyses suggest that routine use of THA in patients over 50 years of age may not confer a clinically meaningful advantage. Any potential benefit of THA compared with HA appears to be small and unlikely to be clinically relevant (50).

A post hoc subgroup analysis of the HEALTH trial evaluated 143 particularly fit patients aged 70 years or younger, all independently mobile and classified as ASA 1 or 2 prior to injury. The mean age in this subgroup was approximately 66 years. No clinically meaningful differences in functional outcomes between THA and HA were observed at 2-year follow-up. In the overall HEALTH trial, 1,495 patients aged over 50 years were randomized (51).

Similarly, an updated meta-analysis of randomized controlled trials including patients over 50 years of age with FNF demonstrated comparable outcomes between THA and HA with respect to revision rates, mortality, dislocation, and functional recovery, with only minimal differences in health-related quality of life favoring THA (52).

### To cement or not to cement?

Gonzalez Della Valle *et al.* analyzed data from the American Joint Replacement Registry and found that cemented fixation was linked to a 37% lower risk of revision and an 87% lower risk of periprosthetic fracture within 90 days (53).

The use of cemented femoral stems in hip fracture is strongly recommended by the AAOS and NICE guidelines (12, 47).

However, some advocates continue to express concerns about cementing implants in elderly patients due to factors such as increased operative time (54), increased costs (54) and increased medical complications (55) associated with cementation. A study by Heckmann *et al.*, which compared cemented versus uncemented THA for FNF, revealed that after adjusting for confounding variables, the cemented group exhibited 1.4-fold increased odds of medical complications (55).

While having a ten-fold risk (2 versus 0.2%) of pulmonary embolism for cemented as compared to cementless arthroplasty, Kheir *et al.* found no difference in mortality between the groups (56).

**Figure 3:**
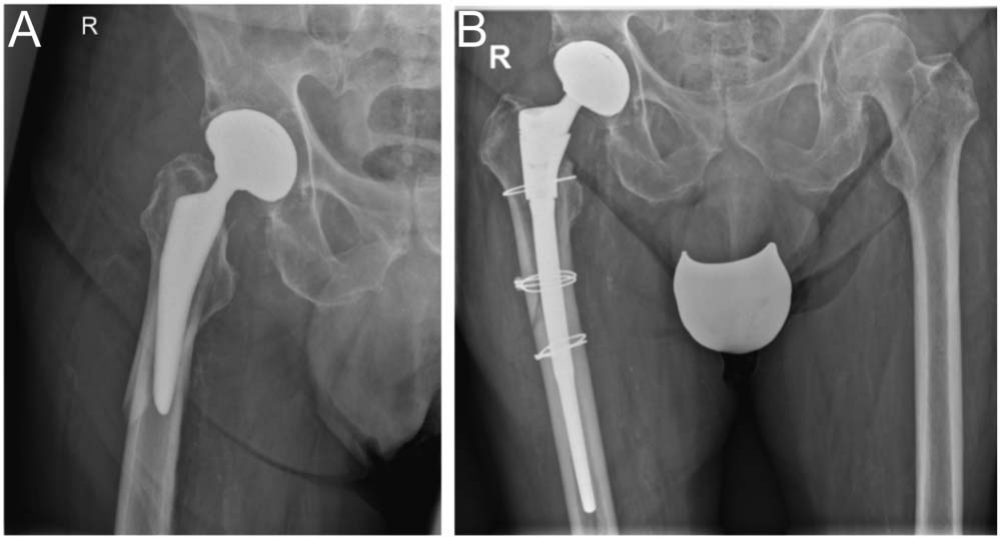
(A) Preoperative and (B) postoperative anteroposterior radiographs of the pelvis of the patient exhibited in Fig. 1, six weeks after index THA surgery for FNF. The patient presents to the emergency department because of right hip pain after a fall from his height. A periprosthetic fracture Vancouver B2 was diagnosed and was treated with a dual mobility revision arthroplasty.

Acknowledging this information, it remains essential to mention that randomized controlled trials demonstrate a higher rate of reoperation for cementless implants compared with cemented ones, primarily due to postoperative periprosthetic fractures (Fig. 3) (57). For this reason, most agree that uncemented implants should be minimized or avoided in FNF altogether (57, 58).

While the advantages of cemented stems are widely accepted in relation to periprosthetic fracture (55), their benefit on quality of life is less clear (59). A randomized control trial demonstrated that patients with cemented HA had a higher quality of life and lower mortality compared to patients with uncemented HA (51, 57, 60).

Fernandez *et al.* reported that cemented HA for FNF resulted in a significantly better quality of life and a lower risk of periprosthetic fracture (0.5 vs 2.1%) compared with uncemented HA (61), while Liu *et al.* reported longer operating time but less pain, lower 1-year mortality, and fewer implant-related complications for cemented HA when compared to uncemented HA (62).

While high-quality evidence supports the use of cemented implants for displaced FNF, which the authors strongly advocate, some discussion persists in the literature regarding the choice between cemented and uncemented stems. In this context, several radiographic indices are used.

**Figure 4:**
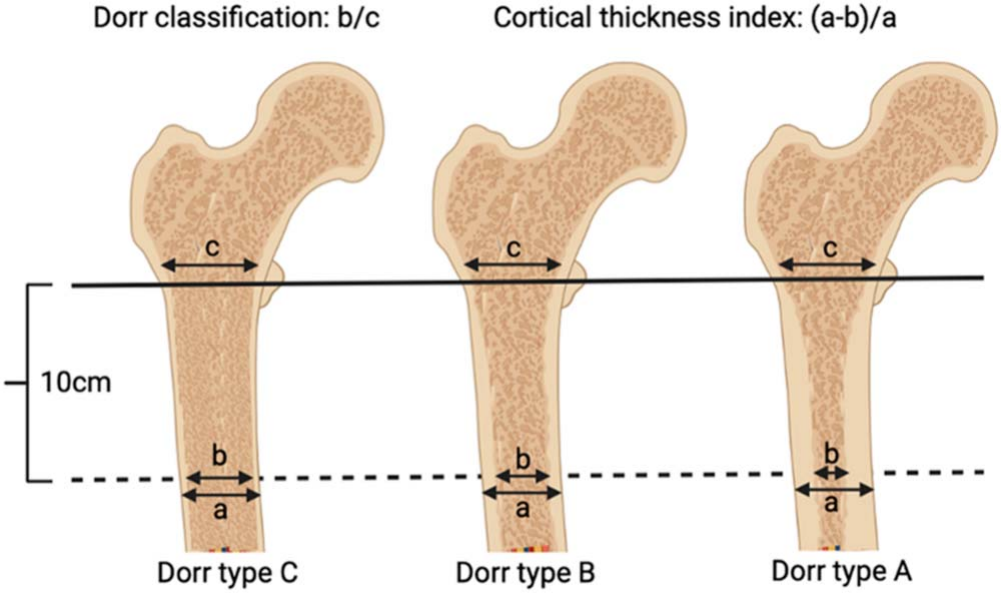
Figure illustrating the measurement of the cortical thickness index (CTI) and the Dorr classification. The CTI is measured by calculating the ratio of the difference between the outer cortical diameter and inner canal diameter to the outer cortical diameter at a point 10 cm distal to the lesser trochanter on an anteroposterior radiograph of the femur. The Dorr classification is determined by evaluating the cortical thickness and medullary canal width on an anteroposterior radiograph of the femur, 10 cm below the lesser trochanter, and categorizing the bone as type A (<0.5), B (0.5–0.75), or C (>0.75) based on the relative thickness of the cortex and shape of the canal. The figure was created using BioRender.com.

The bone morphology can be classified into A, B, and C based on the Dorr classification (63). Kheir *et al.* found that when accounting for confounding variables, Dorr C was significantly associated with increased periprosthetic fractures when performing cementless THA for FNF (56) (Fig. 4).

The Singh index estimates the degree of osteoporosis, on a scale from 1 to 6 based on the trabecular bone pattern of the proximal femur (64). However, because of the poor interobserver correlation and correlation with dual-energy X-ray absorptiometry, its use in daily clinical practice is questioned (65). Another tool is the cortical thickness index (Fig. 4). It can also be measured on standard radiographs, and a lower index is correlated to an increased risk of fracture after HA (66).

**Figure 5:**
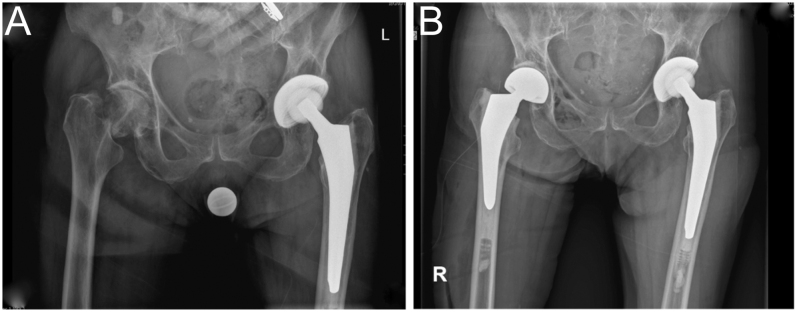
(A) Preoperative and (B) postoperative anteroposterior radiographs of the pelvis of an 86-year-old female who presented with a displaced FNF following a fall from standing height at home. The patient lives at home with assistance and has a Parker score of 5. The cortical thickness index is 0.33, and the femur is classified as Dorr type C. A cemented hemiarthroplasty was implanted.

**Figure 6:**
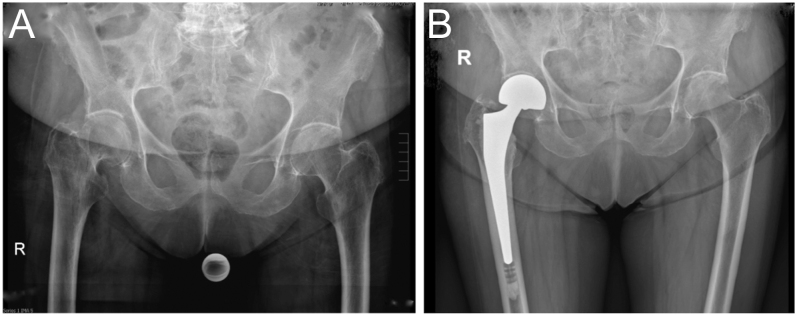
(A) Preoperative and (B) postoperative anteroposterior radiographs of the pelvis of a 90-year-old female who presented with a displaced FNF sustained while grocery shopping. The patient lives alone with her husband and does not require assistance, with a Parker score of 9. The cortical thickness index is 0.53, and the femur is classified as Dorr type B. A cemented hemiarthroplasty was implanted.

While such measures may assist implant selection in the elective arthroplasty setting, FNF in elderly patients are typically osteoporotic fragility fractures and, in the absence of medical contraindications to cementation, should be managed with cemented stems. Although cement-related complications may rarely occur, the overwhelming body of evidence supports the use of cemented implants to optimize fixation and clinical outcomes (Figs 5 and 6).

## Conclusion

There are numerous factors to consider, and while the literature may be controversial, certain recommendations can still be made.

Every effort should be made to perform surgery within 36 h of admission. While we believe that performing surgery at after hours with an experienced team is safe, if expertise is lacking, postponing until the next day to ensure optimal conditions is beneficial.

Given the well-established association between surgical approach and dislocation risk, approaches associated with lower instability rates, such as the anterior or lateral approach, are often recommended in hip fracture arthroplasty, and the posterior approach has been discouraged in the literature due to its higher reported dislocation rates. However, in our view, no single approach is universally superior. Each technique carries specific advantages and disadvantages, and surgeon experience and familiarity with the chosen approach are important determinants of clinical outcomes.

High-quality randomized trials and contemporary meta-analyses have not demonstrated a clinically meaningful advantage of THA over HA, even in relatively fit and independent patients. Therefore, while individualized decision-making remains appropriate, current evidence does not support the routine use of THA solely based on activity level or chronological age.

Although general medical complications related to cementation may occur, their incidence is low compared with the higher risk of periprosthetic fractures associated with cementless stems, thereby supporting the preferential use of cemented fixation.

## ICMJE Statement of Interest

The authors declare that there is no conflict of interest that could be perceived as prejudicing the impartiality of the research reported.

## Funding Statement

This research did not receive any specific grant from any funding agency in the public, commercial or not-for-profit sector.
